# Neuroprotective Effect of Modified Xijiao Dihuang Decoction against Oxygen-Glucose Deprivation and Reoxygenation-Induced Injury in PC12 Cells: Involvement of TLR4-MyD88/NF-*κ*B Signaling Pathway

**DOI:** 10.1155/2017/3848595

**Published:** 2017-10-30

**Authors:** Xu Zhang, Xiaojun Fei, Weiwei Tao, Jingbo Li, Hao Shen, Xuanye Wang, Hongquan Liu, Yun Xu

**Affiliations:** ^1^Jiangsu Province Academy of Traditional Chinese Medicine, Nanjing 210028, China; ^2^Affiliated Hospital of Integrated Traditional Chinese and Western Medicine, Nanjing University of Chinese Medicine, Nanjing 210028, China; ^3^Center for Translational Systems Biology and Neuroscience, Key Laboratory of Integrative Medicine for Brain Diseases, Nanjing University of Chinese Medicine, Nanjing 210023, China; ^4^Nanjing Drum Tower Hospital, Affiliated Hospital of Nanjing University Medical School, Nanjing 210008, China

## Abstract

Modified Xijiao Dihuang (XJDH) decoction has been shown to exert powerful neuroprotective properties in clinical ischemic stroke treatment. It consists of 4 Chinese herbs: Buffalo Horn,* Paeonia suffruticosa* Andrews,* Rehmannia glutinosa* (Gaertn.) DC, and* Paeonia lactiflora* Pall. In the present study, the neuroprotective effect and specific mechanisms of XJDH in protecting PC12 cells from oxygen-glucose deprivation-induced injury were investigated. It was found that OGD/R significantly decreased the cell viability and lactate dehydrogenase (LDH) activity and increased the release of IL-1*β*, IL-6, and TNF-*α* in PC12 cells, and these effects were suppressed by XJDH and one of its major active constituents, paeoniflorin. Additionally, XJDH inhibited caspase-3 activity and reduced cleaved caspase-3 level. Mechanistic studies showed that the expressions of TLR4, MyD88, TRAF6, and NF-*κ*B p65 and phosphorylation of I*κ*B*α* and p65 were significantly lower in the XJDH-treated group than in the OGD/R control group. Additionally, XJDH reversed the OGD/R-induced increases in p-JNK and p-ERK1/2 expression. These results suggest that XJDH protects PC12 cells from oxygen-glucose deprivation-induced injury, which may be associated with the inhibition of the TLR4-MyD88/NF-*κ*B signaling pathway. As an anti-inflammation factor, XJDH might be used as a neuronal protection strategy for the ischemia injury and related diseases.

## 1. Introduction

Ischemic stroke, also known as cerebral infarction, is considered as one of the leading causes of adult mortality and disability worldwide [1]. About 87% of stroke cases are due to sudden occlusion of a blood vessel, which may lead to insufficient oxygen and glucose delivery to support cellular homeostasis and then result in cell death [2, 3]. Therefore, rapid reperfusion in ischemic area for rescuing dying cells is critical in the therapy of cerebral ischemia. However, reperfusion usually causes deterioration of brain injury and a profound inflammatory response [4]. Nowadays few therapeutic options are available for stroke treatment, and thus there is a pressing need for developing new therapeutic strategies.

Although the complex mechanism of cerebra ischemia and reperfusion (I/R) injury has not been exactly demonstrated, the interactions of several pathophysiological processes (i.e., oxygen free radical injury, excitatory amino acid neurotoxicity, and intracellular calcium overload) are shown to be involved in stroke progression. Recent evidences have suggested that innate immune and inflammatory reactions are the main cause contributing to the pathophysiology of cerebral I/R injury [4]. Brain ischemia triggers inflammatory responses and produces cytotoxic substances including TNF, IL-1*β*, iNOS, and other proinflammatory mediators, resulting in more neuronal damage [5]. Anti-inflammatory therapy displayed evident neuroprotective effects in ischemia [6, 7]. Toll-like receptor 4 (TLR4), a germline-encoded pattern recognition receptor expressed by various brain cells, plays an important role in inflammatory responses triggered by I/R injuries [8]. In ischemic/reperfusion condition, TLR4 is activated and leads to recruitment/activation of adaptor protein myeloid differentiation primary response gene (88) (MyD88). Subsequently, the TLR4/MyD88 signaling stimulates TNF receptor-associated factor 6 (TRAF6) and increases the level of nuclear factor kappa B (NF-*κ*B) transcriptional activity and expression of proinflammatory cytokine, thereby triggering inflammatory responses in ischemic brain. Activation of TLR4 also stimulates mitogen-activated protein (MAP) and stress kinases including extracellular-regulated kinase (ERK) 1/2, p38, and Jun kinase (JNK) 1/2, which in turn activates transcription regulators of inflammation. Since inflammatory responses contribute to hypoxic injury and importance of TLR4 in inflammatory mechanisms has been well recognized, blocking TLR4/MyD88 signaling pathway may be a potentially neuroprotective therapeutic strategy for ischemic stroke.

Modified Xijiao Dihuang (XJDH) decoction is derived from a famous ancient Chinese formula originally recorded in “Prescriptions Worth A Thousand Gold,” a medical book written by the “Medicinal King” Sun Simiao (around 700 AD) [9]. The main herbal ingredients of the formula include Buffalo Horn (Shui Niu Jiao in Chinese),* Rehmannia glutinosa *(Gaertn.) DC (Sheng Dihuang in Chinese),* Paeonia lactiflora* Pall. (Shao Yao in Chinese), and* Paeonia suffruticosa* Andrews (Mu Danpi in Chinese). XJDH has been traditionally prescribed for stopping bleeding accompanied with fever, removing toxic substances, and treating spontaneous bleeding, hemoptysis, and nosebleeds [10, 11]. Recently, clinical and preclinical evidences indicated that XJDH and its components possessed neuroprotective effects against neural damage. In a gerbil model of cerebral ischemia/reperfusion, catalpol, a bioactive constituent of XJDH, was observed to significantly improve the stroke index, increase the activity of SOD, and decrease the brain MDA content [12]. As a main component in* Paeonia lactiflora* Pall and XJDH, paeoniflorin was able to reduce cerebral infarct and neurological deficit in ischemia-reperfusion injured rats, which at least in part involved the anti-inflammatory properties [13]. In Lu's work, XJDH was found to improve neurological function deficit in patients with acute cerebral hemorrhage [14]. Although the therapeutic efficiency of XJDH as a neuroprotective agent is attractive, there were no experimental studies to report the effects of XJDH against ischemic stroke injury for all we know. Moreover, the underlying molecular mechanisms of neuroprotective action of this formula remain unclear.

In the present study, we aim to explore whether XJDH eases inflammation and neuron damage injury in PC12 cells after oxygen-glucose deprivation and reoxygenation (OGD/R) and whether the TLR4/MyD88 signaling pathway is related to the potential molecular mechanism.

## 2. Materials and Methods

### 2.1. Chemicals and Antibodies

L-glutamate and 3-(4,5-dimethylthiazol-2-yl)-2,5-diphenyltetrazolium bromide (MTT) were purchased from Sigma Aldrich (St. Louis, MO, USA). Dulbecco's modified Eagle's medium (DMEM), fetal bovine serum (FBS), and other cell culture reagents were purchased from Gibco-Invitrogen (Carlsbad, CA, USA). Antibodies recognizing TLR4, MyD88, TRAF6, NF-*κ*B, cleaved caspase-3, p-ERK, and p-JNK were supplied by Affinity Biosciences. Secondary antibodies were supplied by Proteintech Group Inc. A lactate dehydrogenase (LDH) ELISA Kit was purchased from Shanghai Enzyme-Linked Biotechnology Co., Ltd.

HPLC-grade acetonitrile and formic acid were purchased from ROE Scientific Inc. (USA). Deionized water was purified using a Milli-Q water purification system from Millipore (Bedford, MA, USA). Reference substances including paeoniflorin, albiflorin, and oxypaeoniflorin were purchased from Must Biological Technology Co. Ltd. (Chengdu, China). The purity of each reference compound was over 98% by HPLC.

### 2.2. Preparation of XJDH Decoction

All the crude drugs including 30 g Buffalo Horn, 24 g* Rehmannia glutinosa *(Gaertn.) DC, 12 g* Paeonia lactiflora* Pall., and 9 g* Paeonia suffruticosa *Andrews were purchased from Jiangsu Provincial Hospital of Traditional Chinese Medicine (Jiangsu Province, China). The voucher sample (number 120320) has been deposited in Affiliated Hospital of Integrated Traditional Chinese and Western Medicine, Nanjing University of Chinese Medicine. Firstly, the mixture of XJDH was soaked in 10 volumes of water for 0.5 h and refluxed for 2 h. The water extract was filtered, and the residue was refluxed again with 8 volumes of water for 1 h. The combined filtrations obtained were concentrated into the residues in a vacuum evaporator to produce an extract with concentration of 3 g crude drug/mL. The XJDH extract was stored at –80°C.

### 2.3. High Performance Liquid Chromatography (HPLC) Analysis and Quantification

For HPLC analysis, XJDH was filtered through a 0.45 *μ*m membrane filter before injection. HPLC using Waters 2695 HPLC instrument (Waters Co., Milford, MA, USA) was performed on a SunFire™ C18 column (5 *μ*m, 250 mm × 4.6 mm i.d., Waters Co., Milford, MA, USA) with a mobile phase gradient of acetonitrile-water (2% to 100%) for 70 min. The injection volume was 10 *μ*L of sample and mobile phase flow rate was set at 1 mL/min with UV detection at 254 nm. Acquisition and analysis of chromatographic data were performed using Empower software (Waters Co., Milford, USA). Stock solutions of paeoniflorin, albiflorin, and oxypaeoniflorin were prepared for quantification of XJDH. The concentrations of major constituents were determined by regression equations, calculated in the form of *y* = *ax* + *b*, where *y* and *x* were peak area and contents of the compound, respectively.

### 2.4. Cell Culture

PC 12 cells (rat pheochromocytoma cells) were cultured in DMEM medium supplemented with 5% FBS, 100 U/mL penicillin, and 100 *μ*g/mL streptomycin at 37°C in 5% CO_2_ and a saturated humidified incubator. Before the drug treatment, PC12 cells were adhered and grew in the plate for 24 h.

### 2.5. Oxygen-Glucose Deprivation and Reoxygenation (OGD/R)

Cells were divided into four groups: vehicle control, OGD/R, OGD/R + XJDH, and OGD/R + paeoniflorin. For the vehicle control group, PC12 cells were cultured without OGD/R treatment. For the OGD/R group, the original medium was removed, and then cells were rinsed with PBS (0.01 M, pH 7.4) twice and exposed to DMEM (glucose free) and cultured in a sealed anaerobic chamber flushed with 5% CO_2_ and 95% N_2_ (v/v) for 6 h. XJDH and paeoniflorin were, respectively, added at 30 min before OGD treatment. Then, a reoxygenation period (24 h) was begun by rapidly replacing the DMEM (glucose free) with normal medium and conditions.

### 2.6. Toxicity Assay of XJDH

Cell viabilities of PC12 cells were determined after 24 h of the treatment with various concentrations of XJDH (0–2.5 mg/mL) and paeoniflorin (0–80 *μ*M) according to the MTT method. The absorbance of the samples was quantified at 490 nm using spectrophotometer.

### 2.7. Cell Viability Analysis

PC12 cells were seeded in a density of 5 × 10^4^ cells/mL in 96-well plates and cultured for 24 h. Then, the cells were treated with XJDH (0–2.5 mg/mL) for 30 min and then subjected to OGD damage. After that, OGD was terminated and the cells were further cultured for 24 h with XJDH and paeoniflorin. Cell viability was detected by an MTT assay.

### 2.8. Lactate Dehydrogenase (LDH) Assay

LDH is a stable cytoplasmic enzyme which catalyzes the interconversion of lactate and pyruvate. When the cell plasma membrane is damaged, intracellular LDH is rapidly released into the culture supernatant. In this work, LDH was detected by a colorimetric assay to investigate cytotoxicity. PC12 cells were treated as described in Section 2.3, and then the cells were lysed using Triton X-100 for 20 min at 4°C. After centrifugation, the supernatant was harvested, and LDH was detected by an LDH assay kit (Shanghai Enzyme-Linked Biotechnology Co., Ltd., Shanghai, China) according to the manufacturer's instructions. The absorbance was detected at 450 nm and the relative cell viability was calculated as follows: viability (%) = (OD_treatment_ − OD_treatment  blank_)/(OD_max.   LDH  activity_ − OD_max.   LDH  activity  blank_) × 100%. OD_treatment_ is absorbance of drug-treated group; OD_treatment  blank_ is the background blank absorbance of drug-treated group; OD_max.   LDH  activity_ is absorbance of maximum LDH released group; OD_max.   LDH  activity  blank_ is the background blank absorbance of maximum LDH released group.

### 2.9. Caspase-3 Activity Assay

Caspase-3 activity was detected using caspase-3 ELISA Kit (Shanghai Enzyme-Linked Biotechnology Co., Ltd., Shanghai, China). PC12 cells were seeded on 100 mm dishes for 24 h. After OGD/R treatment as described in Section 2.3, the cells were lysed with lysis buffer (provided by the kit) for 30 min on ice. Then, cell lysis was centrifuged at 10,000*g* for 15 min at 4°C, and the supernatant was harvested. The obtained supernatant was incubated with reaction solution and Ac-LEHD-pNA substrate solution at 37°C for 4 h. Finally, the absorbance was detected at 405 nm and the relative caspase-3 activities were calculated. Relative caspase-3 activity  (%) = (OD_Treatment_ − OD_Treatment  blank_)/(OD_Control_ − OD_Control  blank_) × 100%.

### 2.10. Measurement of TNF-*α*, IL-1*β*, and IL-6 Secretion

The levels of inflammatory mediators such as TNF-*α*, IL-1*β*, and IL-6 in culture medium were determined by ELISA kits (Shanghai Enzyme-Linked Biotechnology Co., Ltd., Shanghai, China) according to the manufacturer's protocols. Concentrations were calculated with reference to the standard curve.

### 2.11. Western Blot Assay

After treatment as described in Section 2.3, the PC12 cells were lysed in RIPA buffer with cocktail protein inhibitors. Cell lysates were separated by 10–12% SDS-PAGE and transferred onto a PVDF membrane (Millipore, Billerica, MA, USA). Then, the membranes were soaked with 5% nonfat milk and individually incubated overnight at 4°C with primary antibodies: TLR4, MyD88, TRAF6, NF-*κ*B, cleaved caspase-3, p-ERK, and p-JNKp38. The blots were washed and developed with enhanced chemiluminescent substrate and imaged using ChemiDoc XRS. The optical density (OD) values of the bands were detected by using Carestream Molecular Imaging Software, and statistical data was adjusted to correspond to internal reference expression for eliminating the variations of protein expression (OD value of target protein versus OD of corresponding internal reference).

### 2.12. Statistical Analysis

All the data were expressed as mean ± standard deviation (SD). One-way ANOVA was used to determine significant differences among all groups. *P* < 0.05 was considered significant.

## 3. Results

### 3.1. HPLC Analysis of XJDH Decoction

**Figure 1:**
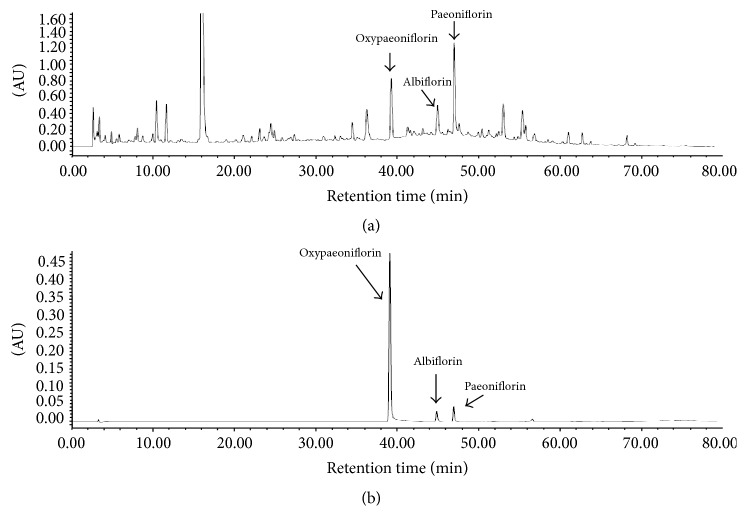
HPLC analysis and quantification of XJDH. HPLC chromatograms of XJDH (a) and oxypaeoniflorin, albiflorin, and paeoniflorin reference (b) obtained using a SunFire™ C_18_ column monitored at 254 nm and eluted with 98% water to 100% acetonitrile for 70 min at a flow rate of 1.0 mL/min.

HPLC analysis was performed to determine the content of three major constituents from XJDH decoction. The mobile phase and elution program were optimized. We found that a mobile phase consisting of acetonitrile and H_2_O at optimized elution program can separate paeoniflorin, albiflorin, and oxypaeoniflorin with full peak-to-baseline resolution (Figure 1). Based on UV maximal absorption, we detected paeoniflorin, albiflorin, and oxypaeoniflorin at 254 nm for quantitative analysis. The contents of paeoniflorin, albiflorin, and oxypaeoniflorin in XJDH were 0.232 ± 0.031%, 0.276 ± 0.018%, and 0.019 ± 0.002%, respectively. Linear calibration curve showed good linear regression (*r*^2^ > 0.999) within test ranges; the LOD (S/N = 3) and the LOQ (S/N = 10) were less than 0.5 and 1.0 *μ*g for paeoniflorin, albiflorin, and oxypaeoniflorin (Table 1).

### 3.2. XJDH Increased Cell Viability after OGD/R

**Figure 2:**
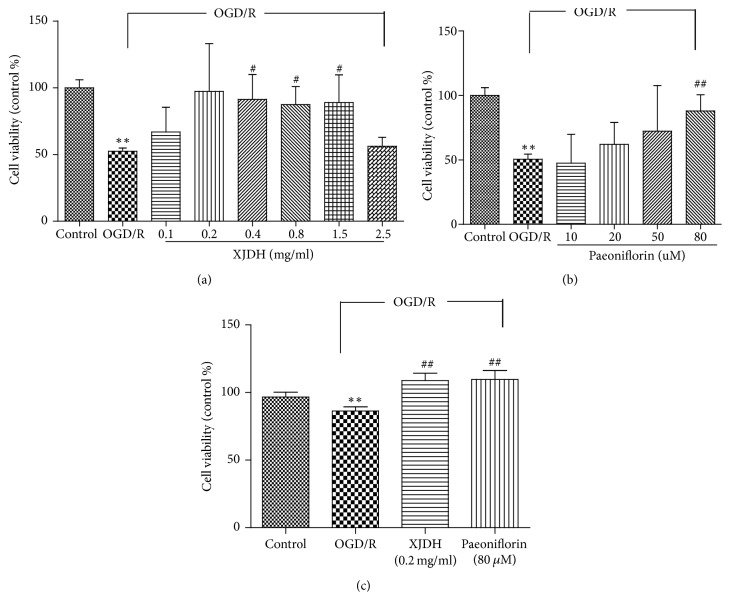
Protective effects of XJDH and paeoniflorin against OGD/R-induced cell death in PC12 cells. Cell viability and toxicity were determined by MTT (a, b) and LDH assay (c). Cells were pretreated with XJDH and for min, followed by exposure to 6 h OGD damage and 24 h reoxygenation. ^*∗∗*^*P* < 0.01 versus control; ^#^*P* < 0.05 and ^##^*P* < 0.01 versus OGD/R-treated cells. All data are represented as the mean ± SD of three independent experiments.

First, we examined whether XJDH and its main components paeoniflorin had a toxic effect on cellular viability. PC12 cells were treated with different concentrations of XJDH and paeoniflorin, and the cell viability was determined using a MTT assay. We found that the cell viabilities of PC12 cells treated with 0.1–1.5 mg/mL XJDH were comparable to that of normally cultured cells, indicating that XJDH did not significantly affect PC12 cell proliferation (data not shown). Similarly, paeoniflorin was not toxic at tested concentrations (0–80 *μ*M). Then, we tested whether XJDH and paeoniflorin could protect PC12 cells against OGD/R injury. As presented in Figure 2(a), the viabilities of PC12 cells decreased to 43.75  ±  2.4% after 6 h OGD and 24 h reperfusion. XJDH at the concentrations of 0.4, 0.2, and 0.1 mg/mL notably restored cell viabilities to 91.9  ±  2.6%, 97.3  ±  3.5%, and 66.9  ±  1.8%. However, XJDH at 2.50 mg/mL caused an obvious decrease in cell survival. Considering a significant neuroprotective effect of XJDH at 0.2 mg/mL, we chose this concentration to complete the following experiments. Paeoniflorin at concentration of 80 *μ*M also significantly attenuated OGD/R-induced cell death (Figure 2(b)).

### 3.3. XJDH Protected PC12 Cells against OGD/R-Induced LDH Release

LDH is a marker widely used to evaluate the damage and toxicity of cells. To further address the protective activity of XJDH on PC12 cells suffering from OGD/R injury, LDH assay was performed. As shown in Figure 2(c), the relative viability of PC12 cells was significantly suppressed after OGD/R (*P* < 0.01, compared with vehicle control). When cells were pretreated with XJDH 0.2 mg/mL and paeoniflorin** (**80 *μ*M**)**, the viability was markedly increased, suggesting an effective neuroprotective effect against OGD/R insult.

### 3.4. XJDH Reduced Inflammatory Cytokine Level in PC12 Cells after OGD/R

**Figure 3:**
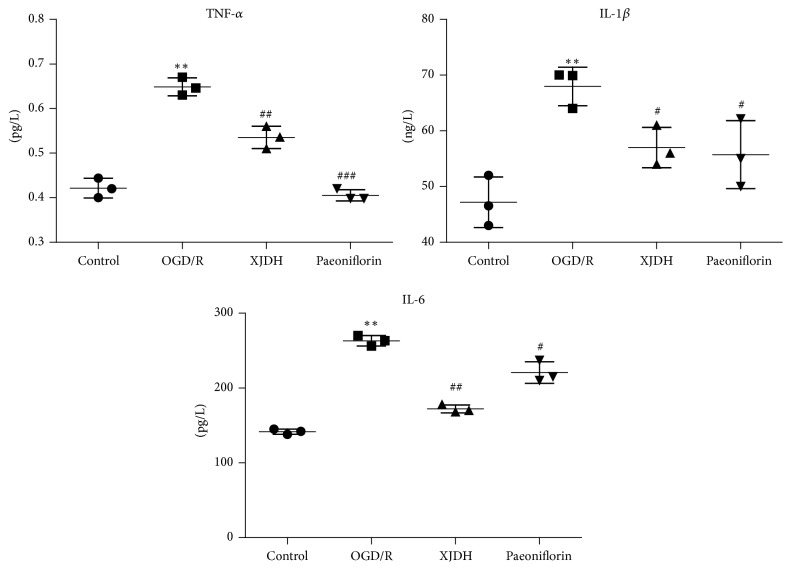
Effects of XJDH and paeoniflorin on IL-1*β*, IL-6, and TNF-*α* secretion in OGD/R-treated PC12 cells. ^*∗∗*^*P* < 0.01 versus control; ^#^*P* < 0.05, ^##^*P* < 0.01, and ^###^*P* < 0.001 versus OGD/R-treated cells. All data are represented as the mean ± SD of three independent experiments.

Inflammatory cytokines such as TNF-*α*, IL-1*β*, and IL-6 play pivotal roles in the pathogenesis of ischemic stroke. In order to investigate the effects of XJDH treatment on the inflammatory response in PC12 cells after OGD/R, we examined the expression levels of TNF-*α*, IL-1*β*, and IL-6 in culture medium. As shown in Figure 3, after 6 h OGD exposure, the levels of IL-1*β*, IL-6, and TNF-*α* in the culture medium were significantly elevated approximately 1.45-fold (*P* < 0.05), 1.85-fold (*P* < 0.05), 1.55-fold (*P* < 0.05) compared with the control group. When pretreated with XJDH and paeoniflorin, IL-1*β*, IL-6, and TNF-*α* secretion induced by OGD were remarkably inhibited (*P* < 0.05 versus OGD), indicating that XJDH and paeoniflorin could decrease inflammatory response after OGD/R damage.

### 3.5. XJDH Inhibited the Expressions of TLR4 and Downstream Signaling Ligand MyD88 after OGD/R

**Figure 4:**
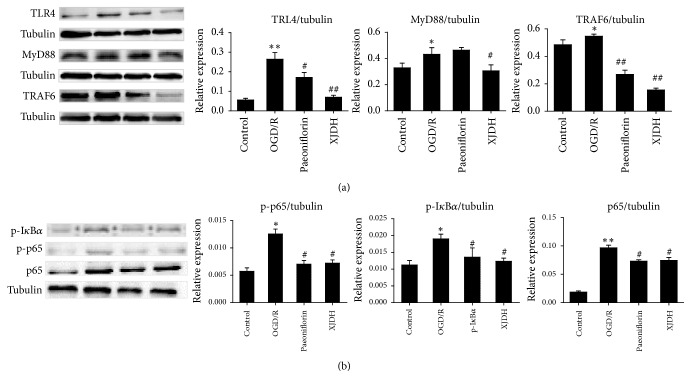
Protective effects of XJDH and paeoniflorin on regulation of proteins in TLR4-MyD88/NF-*κ*B signaling pathway in PC12 cells. Expressions of TLR4, MyD88, and TRAF6 (a) and phosphorylation of I*κ*B*α* and NF-*κ*B p65 (b) were detected by western blots, and tubulin was used as a control. Bar graph represents semiquantitative densitometry from western blot analysis. ^*∗*^*P* < 0.05 and ^*∗∗*^*P* < 0.01 versus control; ^#^*P* < 0.05 and ^##^*P* < 0.01 versus OGD/R-treated cells.

Stimulation of TLR4/MyD88 signaling is important for inflammatory responses in ischemic stroke. It is possible that XJDH inhibits the OGD/R-induced inflammation through regulating TLR4 level. To clarify the neuroprotective mechanisms of XJDH, the effects of XJDH on expression of TLR4 and downstream signaling proteins (Figure 4(a)) were investigated using western blotting. To our expectation, 6 h OGD followed by 24 h reoxygenation notably induced TLR4 expression and the subsequent signaling effectors activation, reflected by the increased levels of MyD88 and TRAF6, (1.34-, 1.15-fold relative to control, resp.). Western blotting analysis showed that TLR4, MyD88, and TRAF6 protein levels in PC12 cells of the XJDH (0.2 mg/mL) pretreatment group were significantly decreased compared with those of the OGD/R group. Additionally, TLR4 level and its downstream proteins were also significantly decreased by paeoniflorin (Figure 4(a)). These results suggested that neuroprotective effect of XJDH against OGD/R-induced injury may be mediated by the regulation of TLR4/MyD88 inflammatory signaling pathways.

### 3.6. XJDH Inhibited NF-*κ*B Activation in PC12 Cells

To further explore mechanisms of XJDH, we investigated effects of XJDH on NF-*κ*B activation in PC12 cells. Total protein was extracted from PC12 cells 24 h after the last drug challenge. The expression levels of NF-*κ*B p65 and I*κ*B*α* were analyzed by western blot analysis. Our data showed that OGD/R stimulation induced the phosphorylation of both p65 and I*κ*B*α* (Figure 4(b)). XJDH (0.2 mg/mL) attenuated the phosphorylation of I*κ*B*α* and p65 (Figure 4(b)). The total NF-*κ*B p65 subunit showed similar patterns as p-p65. These results confirmed that XJDH inhibited TNF-*α* induced NF-*κ*B activation in PC12 cells.

### 3.7. XJDH Had Little Effect on MAPK Pathway in OGD/R-Treated PC12 Cells

**Figure 5:**
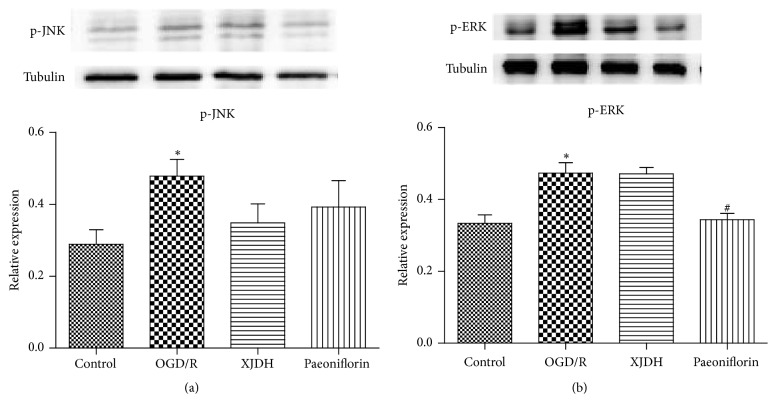
The impact of XJDH and paeoniflorin on protein expression of p-ERK1/2 and p-JNK1/2 in MAPK signaling pathway (a). Bar graph represents semiquantitative densitometry from western blot analysis (b). ^*∗*^*P* < 0.05 versus control; ^#^*P* < 0.05 versus OGD/R-treated cells.

TLR4 activation may stimulate phosphorylation of MAPK pathway. Hence we investigated whether MAPK signaling participated in the neuroprotective effect of XJDH on OGD/R injury. As illustrated in Figures 5(a) and 5(b), PC12 cells exposed to OGD/R showed an increase in the phosphorylation of ERK1/2 and JNK (both at *P* < 0.05). The increased expression levels of p-ERK1/2 and p-JNK induced by OGD/R were reduced and pretreated with 0.2 mg/mL XJDH or 80 *μ*M paeoniflorin. However, there was no significant difference between the OGD/R group and XJDH -treated group. These data suggested that XJDH did not exert its protective effects on PC12 cells through regulating MAPK pathway.

### 3.8. XJDH Inhibited Caspase-3 Activity and Cleaved Caspase-3 Expression in OGD/R-Induced PC12 Cells

**Figure 6:**
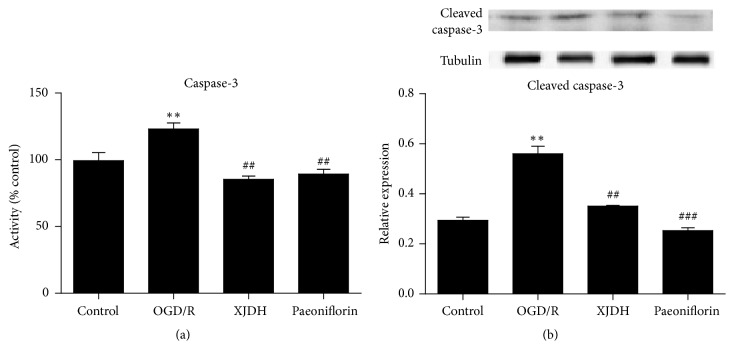
Effects of XJDH and paeoniflorin on caspases-3 activity (a) and cleaved caspase-3 protein expression (b). ^*∗∗*^*P* < 0.01 versus control; ^##^*P* < 0.01 and ^###^*P* < 0.001 versus OGD/R-treated cells.

Caspases-3 played essential roles in apoptosis after cerebral ischemia. In this study, we also investigated the mechanisms by which XJDH and paeoniflorin worked against OGD-induced injury, by analyzing the caspases-3 activity.  Figure 6(a) revealed that OGD induced dramatic increase of caspases-3 activity level compared with vehicle control group. Here, XJDH and paeoniflorin treatment significantly downregulated the caspases-3 activity in PC12 cells. In Figure 6(b), OGD insult induced a significant increase in expression level of the proapoptotic protein cleaved caspase-3, which was inhibited by XJDH and paeoniflorin. These data indicated that caspase-3 apoptosis signal pathway may contribute to the neuroprotective action of XJDH in PC12 cells.

## 4. Discussion

Stroke is one of the leading causes of morbidity and mortality in humans which arises from thrombosis, embolism, or systemic hypoperfusion. Many drugs show neuroprotective effects for stroke treatment, including NMDA receptor antagonist, calcium antagonists, and pituitary adenylate cyclase-activating polypeptide (PACAP) [15, 16]. Nowadays, with advantage of minimal side effects, more and more attention has been focused on constituents from natural medicines or traditional Chinese medicine (TCM) preparations in ischemic stroke therapy [17]. In the present study, we demonstrated that XJDH protected PC12 cells from OGD/R-induced cell damage. OGD/R model is considered as a highly reproducible and appropriate in vitro model of ischemic stroke. By developing OGD/R model in PC12 cells, we found that XJDH administered prior to the insult could prevent the OGD/R-induced cell viability decrease and LDH release. Mechanistic studies showed that the neuroprotective effect of XJDH was performed by inhibiting protein expression of TLR4, MyD88, and TRAF6, NF-*κ*B activation, secretion of TNF-*α*, IL-6, and IL-1*β*, and caspase-3 activity. Activation of TLR4 also stimulates mitogen-activated protein and stress kinases which in turn activate transcription regulators of inflammation. Therefore, we investigated whether MAPK pathway contributed to the neuroprotective effect of XJDH. The results showed that XJDH has little effect on the increased expression of p-ERK and p-JNK triggered by OGD/R exposure. XJDH is a neuroprotective agent that potentially explains the alleviation and prevention from hypoxia-ischemia-induced injury in neurons.

Inflammatory response is a key factor contributing to ischemia-induced injuries and neuronal apoptosis [4]. Cerebral ischemia triggers inflammatory responses and numerous inflammatory mediators that exacerbate ischemic brain injury are induced at the transcriptional level, including enzymes required for prostaglandin synthesis, cytokines (e.g., IL-6 and TNF-*α*), and chemokines [18]. TLR4, an innate and adaptive immune cell receptor of the pathogen recognition receptor family and damage-associated molecular patterns (DAMP) recognition, has increasingly been recognized to be implicated in cerebral I/R injury [19]. Several reports have demonstrated that TLR4 expression was significantly increased in neural tissue after cerebral I/R injury compared to sham groups [8, 20]. The increase of TLR4 expression would activate TRAF6/NF-*κ*B signaling by the MyD88. NF-*κ*B, a key transcription factor of inflammatory cascades, therein initiates the transcription of proinflammatory cytokines (e.g., TNF-*α*, IL-1*β*, and IL-6). Data from human studies suggested the levels of TNF-*α* and IL-6 were positively correlated with infarct volume and poor clinical outcome [21]. Therefore, regulating TLR4/NF-*κ*B signaling pathway following cerebral I/R injury is a viable target for the treatment of cerebral I/R injury. Neurons deficient in TLR4 showed increased resistance and less apoptotic cell death when subjected to glucose deprivation in an* in vitro* model of ischemic conditions [20]. Animal studies also suggested that TLR4 deficiency reduced infarct sizes and improved neurological outcome [22, 23]. Following TLR4 blockade, NF-*κ*B expression was downregulated compared to cerebral I/R injury alone, while the extent of cerebral I/R injury was also reduced [24]. In the present study, our results showed that XJDH at 0.2 mg/mL significantly decreased the levels of TNF-*α*, IL-1*β*, and IL-6 in OGD/R-treated PC12 cells, indicating that XJDH decreased neuroinflammation after OGD/R. In addition, XJDH markedly decreased the expression levels of TLR4, MyD88, TRAF6, and NF-*κ*B p65 and attenuated the phosphorylation of I*κ*B*α* and p65. These results showed that TLR4-MyD88/NF-*κ*B signaling pathway may be involved in the neuroprotective action of XJDH.

Activation of TLR4 also stimulates MAPK pathway, which plays a critical role in I/R injury. Reports have shown that pharmacological ERK and JNK blockade could prevent OGD/R-induced inflammatory response and improved the outcome of ischemic brain injury by downregulating apoptosis signaling [25, 26]. The roles of ERK in the pathophysiology of cerebral ischemia are still controversial. Some reports demonstrated that the phosphorylation of ERK after ischemia might support neuronal damage [27]. In this paper, the effect of XJDH on phosphorylation of ERK and JNK was investigated. Consistent with previous reports, we observed that the levels of p-ERK and p-JNK were persistently increased after OGD/R. The administration of XJDH prevented the increased level of p-ERK and p-JNK to some extent. However, there was no significant difference between the OGD/R-treated group and XJDH-treated group. These data suggested that MAPK pathway may have little contribution to the pharmacological action of XJDH against OGD/R injury.

Caspase-3, a key mediator of apoptosis, is thought to play a central role in cell apoptosis. Active caspase-3 can lead to DNA fragmentation and eventually apoptosis [28]. Studies have showed that activated caspase-3 cleaves proteins that were essential in maintaining neuronal process integrity [29]. Several hours after hypoxia-ischemia, neurons started to undergo caspase-3-dependent apoptosis [30]. In our work, the effect of XJDH on caspases-3 activity and cleaved caspase-3 protein expression were also tested. We found that XJDH significantly decreased caspases-3 activity and the level of cleaved caspases-3 in PC12 cells exposed to OGD/R, suggesting that the action of XJDH against injury by OGD/R could be partly related to its antiapoptotic effects.

**Figure 7:**
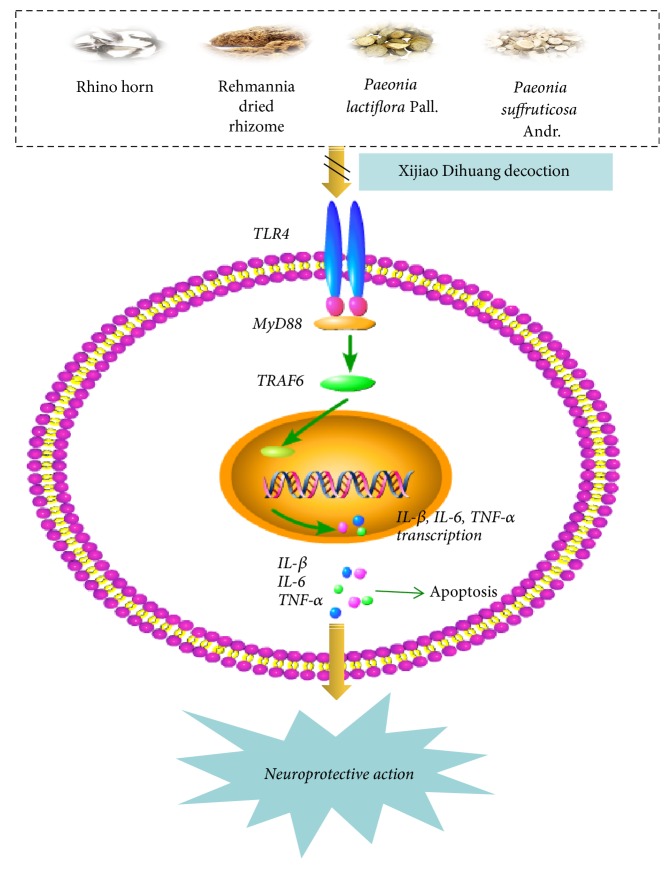
Proposed model for the neuroprotective effects of XJDH in OGD/R injury. XJDH inhibits TLR4/MyD88/NF-*κ*B signaling to ameliorate neuroinflammation and OGD/R injury.

To sum up, the study presented here, for the first time, the fact that herbal medicine XJDH ameliorated the OGD/R-induced damage in PC12 cells, which was associated with its inhibition to TLR4-MyD88/NF-*κ*B pathway and the proinflammatory cytokines (Figure 7). Based on results, we provide a possible explanation and the opportunity for the evaluation of XJDH in the treatment of brain injury. However, more in-depth mechanisms and clinical studies of XJDH against ischemic stroke should be further elucidated for its application in the clinical practice.

## Figures and Tables

**Table 1:** Concentration, calibration curve, regression data, LODs, and LOQs for paeoniflorin, albiflorin, and oxypaeoniflorin by HPLC.

Compounds	Concentration (%)	Calibration curve	*r* ^2^	LOD (*μ*g)	LOQ (*μ*g)
Oxypaeoniflorin	0.019 ± 0.002	*y* = 1*E* + 06*x* + 132000	0.9997	0.02	0.07
Albiflorin	0.276 ± 0.018	*y* = 32176*x* + 211664	0.9993	0.25	0.70
Paeoniflorin	0.232 ± 0.031	*y* = 110720*x* + 42996	0.9994	0.46	0.88
